# Factors Associated With Elevated Sepsis in Obstetrics Scores Among Antenatal Women: An Analytical Study

**DOI:** 10.7759/cureus.111416

**Published:** 2026-06-24

**Authors:** Nidhi Agrawal, Rubina Dohare, Surbhi Tiwari

**Affiliations:** 1 Obstetrics and Gynecology, Government Medical College, Datia, Datia, IND; 2 Obstetrics and Gynecology, People's College of Medical Sciences and Research Center, Bhopal, IND; 3 Obstetrics and Gynecology, Mahaveer Institute of Medical Sciences, Bhopal, IND

**Keywords:** early detection, intrapartum care, maternal sepsis, pregnancy, risk factors, roc curve, sepsis in obstetrics score

## Abstract

Background

Maternal sepsis remains a major cause of maternal morbidity and mortality, particularly in resource-limited settings where early recognition is often delayed. However, conventional sepsis scoring systems have limited applicability in pregnancy because physiological adaptations of gestation can mimic features of sepsis and affect score performance, highlighting the potential role of pregnancy-specific tools, such as the Sepsis in Obstetrics Score (SOS), for risk stratification.

Materials and methods

This analytical cross-sectional study was conducted at a tertiary care center in Bhopal, India, from January 2022 to January 2023. A total of 450 antenatal women with suspected infection were included. The SOS was calculated at admission using pregnancy-adjusted physiological and laboratory parameters and categorized as low risk (SOS <6) or high risk (SOS ≥6), indicating increased risk of severe sepsis-related adverse outcomes. Associations between clinical variables and elevated SOS were assessed using the chi-square test with odds ratios (OR). Continuous variables were compared using the independent samples t-test. Multivariable logistic regression analysis identified independent predictors of elevated SOS.

Results

Among the 450 participants, 50 (11.1%) were categorized as high risk (SOS ≥6). Significant predictors of elevated SOS included ≥4 vaginal examinations, premature rupture of membranes (PROM), PROM lasting >24 hours, and abnormal vaginal discharge. Multivariable analysis identified PROM >24 hours (adjusted OR (aOR) 3.48), ≥4 vaginal examinations (aOR 3.05), and abnormal vaginal discharge (aOR 2.21) as independent predictors. Women with elevated SOS scores demonstrated significant physiological derangements.

Conclusions

The SOS may be useful as a bedside risk-stratification tool among antenatal women with suspected infection. Elevated SOS was primarily associated with modifiable intrapartum and infection-related factors, highlighting potential opportunities for preventive obstetric interventions.

## Introduction

Maternal sepsis remains a significant and largely preventable cause of maternal morbidity and mortality worldwide, contributing to intensive care admissions, organ dysfunction, and death during pregnancy and puerperium. Obstetric infections complicate approximately 7-8% of pregnancies, while confirmed maternal sepsis occurs in up to 0.3% of deliveries, with a considerable proportion progressing to severe disease requiring critical care [1]. Despite improvements in maternal health outcomes, sepsis continues to impose a substantial burden, particularly in resource-limited settings, where delays in recognition and timely management are common.

Early identification of sepsis during pregnancy is challenging because of physiological adaptations, including baseline tachycardia, increased respiratory rate, leukocytosis, and reduced systemic vascular resistance. These changes may overlap with the early manifestations of systemic inflammatory response, thereby limiting the diagnostic accuracy of conventional scoring systems. Standard tools such as the modified Systemic Inflammatory Response Syndrome (SIRS), the Modified Early Warning Score (MEWS), and the quick Sequential Organ Failure Assessment (qSOFA) have demonstrated suboptimal performance in obstetric populations and often fail to reliably distinguish normal physiological changes from early sepsis [2-4]. Although modified obstetric criteria with adjusted thresholds have been proposed, no single scoring system has consistently demonstrated adequate sensitivity and specificity in pregnant women [4].

The Sepsis in Obstetrics Score (SOS), originally developed by Albright et al. [5], is a pregnancy-specific scoring system that incorporates routinely available physiological and laboratory parameters, including maternal temperature, heart rate, respiratory rate, systolic blood pressure, oxygen saturation, white blood cell count, and serum lactate level. Unlike conventional sepsis screening tools, the SOS was specifically designed to account for the physiological adaptations of pregnancy that may influence the interpretation of vital signs and laboratory findings. The SOS was selected for the present study because it relies on readily available clinical and laboratory parameters, can be easily applied in routine obstetric practice, and has demonstrated potential utility for identifying women at increased risk of adverse sepsis-related outcomes. Previous studies have reported that elevated SOS scores are associated with increased risk of adverse maternal outcomes, including intensive care admission and severe infection-related complications [6]. Elevated SOS scores have also been associated with increased maternal mortality and poorer neonatal outcomes. However, variations in optimal cutoff values and diagnostic performance have been reported across different settings [7,8].

Other pregnancy-specific models, including the Sepsis-Associated Adverse Outcomes in Pregnancy (SAAP) score and modified qSOFA-P, have demonstrated moderate predictive accuracy, reflecting ongoing efforts to improve early detection tools in obstetric care [9,10]. However, variability in performance and limited validation across diverse populations highlight the need for further context-specific evaluation, particularly in resource-constrained settings.

In addition to scoring systems, several clinical and intrapartum factors increase the risk of maternal sepsis. Common sources of infection include urinary tract infections, chorioamnionitis, endometritis, and wound infections, with *Escherichia coli* and *Streptococcus* species frequently implicated [11]. Identified risk factors include anemia, cesarean delivery, postpartum hemorrhage, prolonged rupture of membranes, and repeated intrapartum interventions that facilitate ascending infections [12]. Although clinical guidelines emphasize early recognition, prompt antibiotic therapy, and adherence to standardized care bundles, their implementation remains inconsistent in many settings [3,13]. Furthermore, the rise in antimicrobial resistance underscores the importance of timely and appropriate management [14-16].

Although the SOS has been evaluated in selected obstetric populations, evidence regarding its utility in antenatal women with suspected infection in Indian tertiary care settings remains limited. Furthermore, few studies have examined the relationship between elevated SOS categories and potentially modifiable intrapartum practices that may contribute to sepsis risk. Evaluating the performance of the SOS and identifying factors associated with elevated scores may help strengthen risk-based assessment, improve clinical vigilance, and inform preventive obstetric strategies in resource-constrained healthcare settings.

Therefore, the primary objective of the study was to examine factors associated with elevated SOS among antenatal women with suspected infection and to describe the maternal, obstetric, and physiological characteristics associated with higher-risk SOS categories. The study also aimed to assess the association between clinical, obstetric, and intrapartum factors and elevated SOS values, with particular emphasis on identifying modifiable determinants relevant to routine obstetric care.

## Materials and methods

Study design and setting

This hospital-based analytical cross-sectional study was conducted in the Department of Obstetrics and Gynecology at Gandhi Medical College, Bhopal, India, over a period of one year, from January 2022 to January 2023. Data were collected as part of routine clinical care to reflect real-world obstetric practice.

Study population

The study included antenatal women attending the outpatient department or emergency unit or admitted to antenatal wards during the study period. Women presenting with clinical suspicion of infection or systemic inflammatory features, such as fever, tachycardia, tachypnea, or abnormal vaginal discharge, were screened for eligibility. During the study period, 18,085 antenatal women attended the hospital, of whom 450 consecutive eligible participants were included in the final analysis.

Inclusion and exclusion criteria

Pregnant women of any gestational age with suspected infection or systemic inflammatory features were included after obtaining written informed consent. Postpartum women and those with chronic systemic illnesses unrelated to pregnancy (e.g., chronic cardiac or pulmonary disorders) that could independently affect physiological parameters were excluded. Women with incomplete clinical or laboratory data required for SOS calculation were also excluded from the analysis.

Sample size and sampling technique

A total of 450 participants were included using consecutive sampling. All eligible antenatal women presenting during the study period were recruited sequentially. The sample size was determined based on feasibility, expected patient availability during the study period, and prior literature evaluating SOS performance in obstetric populations. Consecutive enrollment was adopted to minimize selection bias and improve representativeness of routine clinical practice.

Variables and measurements

The SOS framework used in this study was adapted from the literature for academic and non-commercial research purposes, with appropriate citation to the original development study by Albright et al. [5]. No copyrighted instrument was reproduced verbatim, and no modification requiring formal licensing was undertaken.

The SOS is a pregnancy-specific scoring system that incorporates physiological and laboratory parameters adjusted for normal gestational changes, including maternal temperature, heart rate, respiratory rate, systolic blood pressure, oxygen saturation, white blood cell count, and serum lactate level. Higher scores indicate greater physiological derangement and increased risk of severe sepsis-related adverse outcomes.

Based on previously published validation studies, an SOS ≥6 has been associated with increased risk of severe sepsis-related adverse outcomes and need for intensive monitoring. Therefore, elevated SOS was defined as SOS ≥6 for the present study. Because of the cross-sectional design, actual progression to severe sepsis, organ dysfunction, ICU admission, septic shock, or mortality was not prospectively assessed. Consequently, the primary study outcome was an elevated SOS category (SOS ≥6) as a marker of increased risk rather than confirmed severe sepsis itself.

Participants were categorized into a low-risk group (SOS <6) and a high-risk group (SOS ≥6). Sociodemographic variables included age, residence, socioeconomic status, and booking status. Obstetric variables included parity, gestational age, antenatal visits, anemia, hypertensive disorders, gestational diabetes, urinary tract infection, and respiratory infection. Intrapartum variables included number of vaginal examinations, premature rupture of membranes (PROM), PROM lasting >24 hours, prolonged labor, and duration of hospital stay. Clinical parameters, including temperature, pulse rate, respiratory rate, systolic blood pressure, and oxygen saturation, and laboratory parameters, including white blood cell count and serum lactate levels, were recorded at admission.

Data collection

Data were collected using a pretested structured proforma. After obtaining informed consent, demographic, clinical, and obstetric details were documented. Vital signs and laboratory values were recorded, and the SOS was calculated at admission. Data quality was ensured through verification and cross-checking of entries prior to analysis.

Statistical analysis

Data were entered into Microsoft Excel (Microsoft Corp., Redmond, WA, USA) and analyzed using Jamovi version 2.6.44 (The Jamovi Project, Sydney, Australia). Continuous variables were expressed as mean ± standard deviation (SD), while categorical variables were presented as frequencies and percentages. Associations between categorical variables and elevated SOS categories were assessed using the chi-square test. Odds ratios (ORs) with 95% confidence intervals (CIs) were calculated. Continuous variables were compared using the independent samples t-test. Variables demonstrating clinical relevance and/or a p-value <0.20 in bivariate analysis were considered for inclusion in the multivariable model, and adjusted OR (aOR) with corresponding 95% CI were reported. A p-value <0.05 was considered statistically significant.

Ethical considerations

Ethical approval was obtained from the Institutional Ethics Committee of Gandhi Medical College, Bhopal (approval no.: 102/IEC/2021). Written informed consent was obtained from all participants prior to enrollment. Confidentiality was maintained throughout the study, and all data were anonymized before analysis.

## Results

A total of 450 antenatal women were included, of whom 50 (11.1%) were categorized as high risk (SOS ≥6) and 400 (88.9%) as low risk (SOS <6). The mean age of participants was 26.8 ± 4.5 years. Women in the high-risk group had a higher mean age than those in the low-risk group (28.2 ± 4.8 vs. 26.5 ± 4.4 years). Most participants belonged to the 25-30-year age group (42.2%). A higher proportion of high-risk women were from rural areas (70.0% vs. 58.8%) and were multigravida (70.0% vs. 51.2%). The mean gestational age was higher in the high-risk group (36.1 ± 4.1 weeks) compared to the low-risk group (34.2 ± 3.7 weeks) (Table 1).

**Table 1:** Baseline demographic and obstetric characteristics of study participants stratified by SOS category Data are presented as mean ± SD for continuous variables and frequency (percentage) for categorical variables. Participants were categorized into low-risk (SOS <6) and high-risk (SOS ≥6) groups based on SOS at admission. Age is presented both as a continuous variable and as categories based on clinically relevant thresholds. Parity is classified as primigravida and multigravida. SOS: Sepsis in Obstetrics Score, SD: standard deviation

Variable	Total (n = 450)	SOS <6 (n = 400)	SOS ≥6 (n = 50)
Age (years)	26.8 ± 4.5	26.5 ± 4.4	28.2 ± 4.8
<25 years	180 (40.0)	165 (41.3)	15 (30.0)
25-30 years	190 (42.2)	165 (41.3)	25 (50.0)
>30 years	80 (17.8)	70 (17.5)	10 (20.0)
Residence			
Rural	270 (60.0)	235 (58.8)	35 (70.0)
Urban	180 (40.0)	165 (41.2)	15 (30.0)
Parity			
Primigravida	210 (46.7)	195 (48.8)	15 (30.0)
Multigravida	240 (53.3)	205 (51.2)	35 (70.0)
Gestational age (weeks)	34.6 ± 3.8	34.2 ± 3.7	36.1 ± 4.1

Several intrapartum and infection-related factors were significantly associated with elevated SOS. Women undergoing ≥4 vaginal examinations had higher odds of elevated SOS (OR 2.88; 95% CI: 1.52-5.45; p = 0.002). PROM was also associated with increased risk (OR 2.34; p = 0.007), with a stronger association observed for PROM lasting >24 hours (OR 3.21; p < 0.001). Abnormal vaginal discharge was significantly associated with elevated SOS (OR 2.05; p = 0.011). Anemia showed a borderline association (p = 0.067), whereas prolonged labor was not significantly associated (p = 0.462) (Table 2).

**Table 2:** Association of maternal and intrapartum risk factors with elevated SOS Associations between selected risk factors and elevated SOS were assessed using a chi-square test. Results are presented as crude ORs with corresponding 95% CIs. OR represents the odds of elevated SOS among exposed versus non-exposed groups. *p < 0.05, **p < 0.01, ***p < 0.001. SOS: Sepsis in Obstetrics Score, ORs: odds ratios, CIs: confidence intervals

Risk factor	SOS <6, n (%)	SOS ≥6, n (%)	OR	95% CI	p-value
Anemia	240 (60.0)	35 (70.0)	1.54	0.97-2.45	0.067
≥4 vaginal examinations	120 (30.0)	30 (60.0)	2.88	1.52-5.45	0.002**
PROM	140 (35.0)	28 (56.0)	2.34	1.26-4.36	0.007**
PROM >24 hours	60 (15.0)	18 (36.0)	3.21	1.78-5.79	<0.001***
Prolonged labor	100 (25.0)	15 (30.0)	1.28	0.66-2.47	0.462
Abnormal vaginal discharge	110 (27.5)	24 (48.0)	2.05	1.18-3.55	0.011*

Multivariable logistic regression analysis identified PROM >24 hours as the strongest independent predictor of elevated SOS (aOR 3.48; p < 0.001), followed by ≥4 vaginal examinations (aOR 3.05; p = 0.001) and abnormal vaginal discharge (aOR 2.21; p = 0.009). Anemia was not statistically significant after adjustment (aOR 1.63; p = 0.081) (Table 3).

**Table 3:** Multivariable logistic regression analysis identifying independent predictors of elevated SOS Model fit statistics: AIC = 298.4, McFadden R² = 0.34, Nagelkerke R² = 0.41, Hosmer-Lemeshow p = 0.48 Multivariable logistic regression analysis was performed with SOS ≥6 as the dependent variable. Results are expressed as β coefficients (log-odds), SE, aOR, and 95% CI. Variables included in the model were selected based on clinical relevance and/or p < 0.20 in bivariate analysis. Model performance was evaluated using AIC for model fit, McFadden’s R² and Nagelkerke R² for explanatory power, and the Hosmer-Lemeshow goodness-of-fit test for calibration. A non-significant Hosmer-Lemeshow test (p > 0.05) indicates adequate model fit. SE: standard error, aOR: adjusted odds ratio, CI: confidence interval

Predictor	β	SE	aOR	95% CI	p-value
≥4 vaginal examinations	1.11	0.33	3.05	1.59-5.84	0.001**
PROM >24 hours	1.25	0.31	3.48	1.92-6.31	<0.001***
Abnormal vaginal discharge	0.79	0.30	2.21	1.21-4.02	0.009**
Anemia	0.49	0.28	1.63	0.94-2.83	0.081

A comparison of clinical and laboratory parameters revealed significant physiological differences between the groups. Women with SOS ≥6 had higher temperature (38.9 ± 1.1°C vs. 37.9 ± 1.3°C; p < 0.001), pulse rate (134 ± 29 vs. 121 ± 31 beats/min; p < 0.001), and respiratory rate (36 ± 9 vs. 28 ± 11 breaths/min; p < 0.001). They also had higher white blood cell counts (25,200 vs. 20,300 cells/mm³; p < 0.001) and serum lactate levels (4.0 vs. 3.5 mmol/L; p = 0.004). Oxygen saturation (88.9% vs. 91.6%; p < 0.001) and systolic blood pressure (123 vs. 129 mmHg; p = 0.021) were significantly lower in the high-risk group (Table 4).

**Table 4:** Comparison of clinical and laboratory parameters between low-risk and high-risk groups Continuous variables are presented as mean ± SD and compared using the independent samples t-test. Effect size is reported using Cohen’s d. *p < 0.05, **p < 0.01, ***p < 0.001. SpO₂: peripheral oxygen saturation, WBC: white blood cell count, BP: blood pressure, SD: standard deviation

Variable	SOS <6, mean ± SD	SOS ≥6, mean ± SD	p-value	Cohen’s d
Temperature (°C)	37.9 ± 1.3	38.9 ± 1.1	<0.001***	0.85
Pulse (beats/min)	121 ± 31	134 ± 29	<0.001***	0.43
Systolic BP (mmHg)	129 ± 24	123 ± 25	0.021*	0.24
Respiratory rate	28 ± 11	36 ± 9	<0.001***	0.80
SpO₂ (%)	91.6 ± 4.8	88.9 ± 4.2	<0.001***	0.60
WBC count	20,300 ± 10,200	25,200 ± 8,100	<0.001***	0.52
Serum lactate	3.5 ± 1.4	4.0 ± 1.3	0.004**	0.37

## Discussion

The present study evaluated factors associated with elevated SOS among antenatal women with suspected infection and examined the maternal, intrapartum, and physiological characteristics associated with higher-risk SOS categories. Among the 450 participants, 50 women (11.1%) were categorized as high risk (SOS ≥6), indicating that approximately one in nine women had elevated SOS values suggestive of increased risk for adverse sepsis-related outcomes. The findings indicate that elevated SOS was predominantly associated with intrapartum and infection-related factors rather than baseline maternal characteristics. In the present analysis, anemia showed only a borderline association and did not remain statistically significant after adjustment, suggesting a limited independent role in SOS elevation. In contrast, intrapartum factors demonstrated strong and consistent associations. Women undergoing four or more vaginal examinations had significantly higher odds of elevated SOS, and PROM, particularly beyond 24 hours, emerged as a major determinant. Abnormal vaginal discharge was also independently associated with elevated SOS, indicating the contribution of active genital tract infection. Multivariable logistic regression confirmed that PROM >24 hours was the strongest independent predictor, followed by repeated vaginal examinations and abnormal vaginal discharge, underscoring the importance of modifiable intrapartum practices in relation to elevated sepsis risk.

These findings are consistent with existing evidence linking ascending infections and obstetric interventions to maternal sepsis. Chorioamnionitis and intra-amniotic infection have been well documented as major contributors to maternal and neonatal infectious morbidity, with studies demonstrating substantially increased risks of infection-related complications in affected women and newborns [16]. Systematic reviews from low-resource settings have identified frequent vaginal examinations, prolonged labor, and membrane rupture as important determinants of maternal sepsis [17]. Similarly, evidence from Ethiopia has shown that prolonged rupture of membranes and repeated vaginal examinations significantly increase the risk of puerperal sepsis [18]. The direction and magnitude of associations observed in the present study, particularly for PROM >24 hours (aOR 3.48) and ≥4 vaginal examinations (aOR 3.05), are consistent with these reports and support the biological plausibility that repeated instrumentation and prolonged exposure of the genital tract facilitate ascending infection and systemic inflammatory responses.

In contrast to some epidemiological studies, baseline maternal conditions such as anemia did not demonstrate a strong independent association with elevated SOS after adjustment. Although population-based analyses have identified anemia and other maternal comorbidities as contributors to maternal sepsis [18], such studies frequently focus on clinically confirmed severe sepsis, postpartum infection, or maternal mortality. The current study assessed elevated SOS as a marker of increased sepsis-related risk among antenatal women and therefore may capture earlier physiological changes rather than established disease. Additionally, although a greater proportion of rural and multigravida women were observed in the high-risk group, these variables did not emerge as independent predictors in the final multivariable model, suggesting that immediate clinical and intrapartum factors may exert a stronger influence on SOS elevation.

Broader literature suggests that lower socioeconomic status and rural residence may increase the risk of maternal sepsis because of delayed healthcare access, referral barriers, and disparities in obstetric care [19]. However, these variables were not independently associated with elevated SOS in the present analysis, indicating that their effects may be mediated through other clinical or healthcare-related factors.

Women with elevated SOS exhibited significant physiological abnormalities, including elevated temperature, tachycardia, tachypnea, leukocytosis, elevated serum lactate, and lower oxygen saturation and systolic blood pressure. These findings are consistent with physiological disturbances commonly observed in maternal sepsis and support the construct validity of elevated SOS as a marker of physiological instability [20]. The presence of moderate-to-large effect sizes for key parameters, particularly temperature and respiratory rate, further reinforces the score's clinical relevance in identifying women with greater physiological derangement.

Biomarker research has explored adjunctive tools such as procalcitonin for the identification of maternal infection; however, pregnancy-specific reference ranges remain variable, and evidence supporting routine clinical use remains limited [21]. Consequently, bedside physiological assessment tools such as the SOS may provide a practical, immediately applicable approach to identifying women who warrant closer clinical assessment and monitoring.

As the primary outcome of the present study was elevated SOS (SOS ≥6), the analysis focused on factors associated with higher-risk SOS categories rather than on prediction of clinically confirmed severe sepsis outcomes. Previous validation studies have demonstrated the prognostic utility of SOS for predicting adverse maternal outcomes, including intensive care unit admission, organ dysfunction, and severe sepsis [13]. Although these outcomes were not directly assessed in the present study, the association between elevated SOS and significant physiological abnormalities supports the clinical relevance of SOS-based risk stratification in antenatal women with suspected infection. Kumari et al. reported that elevated SOS scores were associated with greater clinical severity and increased need for intensive monitoring [6]. At the same time, Stephens et al. demonstrated an association between elevated SOS and adverse maternal outcomes [7]. Collectively, these studies support the potential utility of SOS as a structured clinical assessment tool for identifying women who may require enhanced monitoring and timely intervention.

Other pregnancy-specific early warning systems have demonstrated variable performance across obstetric populations. Modified sepsis screening tools may improve early recognition but can also increase false-positive classifications [22]. In this context, the SOS offers several practical advantages, including reliance on routinely available physiological and laboratory parameters and ease of implementation within routine obstetric care without substantial additional resource requirements.

System-level evidence further supports the importance of structured risk assessment in obstetric populations. Findings from the WHO Global Maternal Sepsis Study indicate that infection-related adverse maternal outcomes occur more frequently in resource-limited and non-urban settings, often because of delays in recognition and treatment [23]. The present findings reinforce the need for systematic assessment approaches that may facilitate earlier identification of women at increased risk and support timely clinical decision-making in high-volume obstetric settings.

Overall, the findings suggest that elevated SOS is primarily associated with modifiable intrapartum practices and early indicators of infection rather than chronic maternal conditions. The strong associations observed for prolonged rupture of membranes, repeated vaginal examinations, and abnormal vaginal discharge highlight potentially preventable factors that may contribute to increased sepsis-related risk. The association between elevated SOS and significant physiological abnormalities further supports the clinical relevance of SOS-based risk stratification and its potential to identify antenatal women who may benefit from closer monitoring and clinical evaluation.

Strengths

The present study has several important strengths. It included a relatively large sample of antenatal women (n = 450), including a clearly defined subgroup with elevated SOS, which enhanced the robustness of the statistical analyses. The use of consecutive sampling facilitated the inclusion of real-world clinical cases and helped minimize selection bias. Data were collected using a pretested structured proforma with verification procedures, thereby improving data quality and reliability.

The study comprehensively evaluated demographic, obstetric, intrapartum, clinical, and laboratory characteristics, allowing a multidimensional assessment of factors associated with elevated SOS. The use of multivariable logistic regression enabled the identification of independent predictors while accounting for potential confounding variables. Additionally, comparing physiological and laboratory parameters across SOS risk categories provided valuable insights into the clinical characteristics associated with elevated scores.

Importantly, the findings reflect routine obstetric practice in a tertiary care setting, enhancing their relevance and applicability to similar healthcare environments, particularly in resource-constrained settings where timely identification of women at increased risk of sepsis-related complications is essential.

Limitations

This study has certain limitations that should be considered while interpreting the findings. First, the hospital-based cross-sectional design precludes establishing temporal or causal relationships between identified risk factors and elevated SOS. Second, the study was conducted at a single tertiary care center, which may limit the generalizability of the findings to other healthcare settings, particularly primary and rural healthcare facilities.

Although consecutive sampling was employed to reduce selection bias, only antenatal women presenting with suspected infection or systemic inflammatory features were included, potentially resulting in an overrepresentation of higher-risk individuals. The SOS was assessed only at admission, and serial measurements were not performed; therefore, changes in clinical status over time could not be evaluated.

Microbiological confirmation of infection was not uniformly available, raising the possibility of misclassification based on clinical criteria alone. Additionally, some comorbid conditions and obstetric complications may have lacked sufficient statistical power to detect significant associations due to relatively small subgroup sizes. Residual confounding from unmeasured factors, including healthcare-seeking behavior, referral delays, access to care, and quality of intrapartum management, cannot be excluded.

An important limitation is that the primary outcome was elevated SOS rather than clinically confirmed severe sepsis, organ dysfunction, intensive care unit admission, septic shock, or maternal mortality. Consequently, the study evaluated factors associated with elevated SOS and SOS-based risk stratification, rather than directly predicting adverse maternal outcomes. Prospective longitudinal studies are needed to determine the prognostic value of SOS for clinically confirmed sepsis-related outcomes.

Clinical implications

The findings of this study have important clinical implications. The strong associations observed between elevated SOS and modifiable intrapartum factors, particularly repeated vaginal examinations, prolonged rupture of membranes, and abnormal vaginal discharge, highlight potential opportunities for preventive intervention. Limiting unnecessary vaginal examinations, ensuring adherence to aseptic techniques, and timely management of membrane rupture may help reduce infection-related risk among antenatal women.

Because the SOS is based on routinely available physiological and laboratory parameters, it can be readily incorporated into obstetric assessment without requiring specialized investigations or additional resource-intensive procedures. The score may assist clinicians in identifying women with greater physiological derangement who may benefit from closer observation, further evaluation, and timely clinical intervention.

Integration of SOS-based assessment into routine antenatal and intrapartum care may strengthen risk-based clinical decision-making and support earlier recognition of women who require enhanced monitoring. Implementation should be supported by multidisciplinary collaboration involving obstetricians, nurses, midwives, infection-control personnel, laboratory services, and referral teams. Regular staff training, adherence to infection-prevention practices, and standardized assessment protocols may further improve the identification and management of women at increased risk.

Future prospective longitudinal studies are warranted to validate these findings and determine whether SOS-guided monitoring and management strategies translate into improved maternal outcomes.

## Conclusions

This study suggests that the SOS may be a useful bedside tool for risk stratification among antenatal women with suspected infection. Elevated SOS values were primarily associated with modifiable intrapartum and infection-related factors, particularly prolonged rupture of membranes, repeated vaginal examinations, and abnormal vaginal discharge. Women classified as high risk (SOS ≥6) exhibited significant physiological abnormalities consistent with increased clinical instability. These findings support the potential utility of SOS for identifying women who may require closer monitoring and further clinical assessment in routine obstetric practice. However, because the study evaluated elevated SOS rather than clinically confirmed severe sepsis outcomes, prospective longitudinal studies are required to determine whether SOS-guided assessment can predict adverse maternal outcomes and improve clinical care.
